# Intent to Accept a Valley Fever Vaccine for Humans and Dogs and Factors Influencing Intended Uptake: A Cross-Sectional Survey in Two Endemic Regions

**DOI:** 10.3390/jof12060420

**Published:** 2026-06-10

**Authors:** Julia N. Hermann, Sophia E. Kruger, Natalie Wodniak, Jammie Holland, Veronica Janosick, Asley Sanchez, Julio C. Zuniga-Moya, Dana Brucker, Bianca Torres, Keny Mendoza Melo, Emilse Oliveros, Rasha Kuran, Carlos D’Assumpcao, Royce H. Johnson, R. Scott Van Pelt, Abinash Bhattachan, Abram L. Wagner, Jennifer R. Head

**Affiliations:** 1 Department of Epidemiology, School of Public Health, University of Michigan, Ann Arbor, MI 48109, USA; jnherm@umich.edu (J.N.H.); sophik@umich.edu (S.E.K.);; 2 Clinical Research Institute, Texas Tech University Health Sciences Center, Odessa, TX 79763, USA; 3 School of Medicine, Washington University, St. Louis, MO 63110, USA; 4 Valley Fever Institute, Kern Medical, Bakersfield, CA 93306, USA; 5 Department of Medicine, David Geffen School of Medicine, University of California Los Angeles, Los Angeles, CA 90095, USA; 6 Division of Infectious Disease, Department of Medicine, Kern Medical, Bakersfield, CA 93306, USA; 7 Agricultural Research Service, U.S. Department of Agriculture, Big Spring, TX 79720, USA; 8 Department of Geosciences, Texas Tech University, Lubbock, TX 79409, USA; 9 Institute for Global Change Biology, University of Michigan, Ann Arbor, MI 48109, USA

**Keywords:** coccidioidomycosis, Valley fever, immunization, vaccines, vaccine willingness, dog vaccination

## Abstract

As Valley fever (coccidioidomycosis) vaccine candidates progress towards human trials, a challenge to their eventual introduction is understanding vaccine demand and addressing vaccine hesitancy. We assessed intent to accept a Valley fever vaccine for humans and dogs in two populations living in highly endemic regions: employees at Kern Medical (KM) in Bakersfield, California (N = 103) and members of the public in West Texas (N = 230). We compared the weighted proportions of each population willing to vaccinate themselves and their dogs by demographic and coccidioidomycosis risk factors and assessed the importance of vaccine-related factors on vaccine uptake in each population. We found that 42% (95% confidence interval (CI): 34–49%) of West Texas residents and 76% (95% CI: 63–85%) of KM employees were willing to accept a coccidioidal vaccine, while 49% (95% CI: 41–58%) of West Texas residents and 74% (95% CI: 58–86%) of KM employees were willing to vaccinate their dogs. Among West Texas residents, vaccination willingness was significantly higher among those with prior awareness of Valley fever (adjusted odds ratio (aOR): 3.83, 95% CI: 1.73, 8.49). Across both study populations, absence of side effects was the most important condition that would increase vaccination willingness. Our results indicate substantial interest in a Valley fever vaccine while suggesting that increased Valley fever awareness and minimal vaccine side effects may be important for increased uptake.

## 1. Introduction

Coccidioidomycosis, or Valley fever, is a fungal disease endemic to parts of the southwestern United States. It affects humans and other mammalian hosts through the inhalation of fungal spores, which may be released into the air through wind, dust storms, or soil disturbance [1,2,3]. Approximately 40% of infections are symptomatic, and the most common presentation of disease is respiratory infection or pneumonia, with a small proportion (1–5%) of individuals developing severe, disseminated infection [1].

In highly endemic areas, including southwestern Arizona and the Central Valley and Central Coast regions of California, coccidioidomycosis incidence can exceed 200 cases per 100,000 population, contributing to a high burden on health systems [4]. An estimated 206,000–360,000 symptomatic cases occur annually, yet cases are underreported and underdiagnosed [5] and the disease is not reportable to public health surveillance in all endemic states. As a result, there is limited information available on the burden of disease in several endemic states, including Texas, where Valley fever is understudied and only reportable in El Paso County [1,6,7]. Severe infections may result in chronic infection that often requires lifetime treatment [8]. Beyond its potential burden, the disease is costly. An analysis in Arizona estimated that the average lifetime healthcare costs for individuals diagnosed with Valley f ever exceeded $23,000 for individuals with uncomplicated pneumonia and $1.26 million for those with disseminated infection [9].

*Coccidioides* spp. can also infect domestic dogs. A study in Arizona showed that dogs had a 28% probability of infection by 2 years of age [10]. Infections among dogs tend to geographically overlap within infections in humans. Dog deaths have been reported alongside human outbreaks of Valley fever [11], and analysis of *Coccidioides* spp. serologic data from dogs identified several counties of West Texas in addition to known endemic regions of Arizona and California as hot spots for seropositive dogs [12].

Due to the high incidence of Valley fever in endemic areas, predicted expansion in its geographic endemic range under climate change [13,14], and its high burden on health systems [9], there has been interest in developing a vaccine to prevent pneumonia-like symptoms caused by Valley fever [15,16,17]. As infection with *Coccidioides* spp. is widely considered to be protective against subsequent infections, Valley fever is a rational disease to target for vaccine development [1,17]. While multiple coccidioidomycosis vaccine candidates have been explored to varying degrees, to date no candidate has progressed through Phase III trials in humans and been proven effective [17]; the last candidate to reach Phase III trials in the 1980s was not found to lessen disease severity, which remains a goal for new-age vaccine candidates [18,19]. In 2022, the National Institute of Allergy and Infectious Diseases published a strategic plan to support research and development of human coccidioidomycosis vaccine candidates that can reach clinical trials by 2030, motivating continued research [20]. Recently, a live-attenuated vaccine candidate was shown to be safe in animal studies [21] and efficacious against clinically relevant coccidioidomycosis in a challenge trial among dogs [19]. This vaccine is considered to be a strong candidate for human trials [19].

As Valley fever vaccine candidates progress towards Phase I trials in humans and show promise to become the first vaccine for a fungal infection, understanding demand for the vaccine and identifying and addressing vaccine hesitancy is a key anticipated challenge [15]. In this study, we explored the intent to accept a Valley fever vaccine for humans and dogs in two endemic areas and across two population types: (1) employees at Kern Medical (KM) in Bakersfield, California, where the burden of disease is among the highest in the U.S. [22] and knowledge of the disease and its risk factors is high; and (2) the general population in West Texas, where Valley fever is endemic but not reportable [23], and awareness is believed to be low [6]. Through cross-sectional surveys in these two populations, we aimed to assess factors influencing future acceptance of a Valley fever vaccine in states across a wide range of public awareness of the disease.

## 2. Materials and Methods

### Ethics Statement

Ethical approval for data collection in Bakersfield, CA was obtained from Kern Medical’s Institutional Review Board (IRB) (#24083) in January 2025, and approval for secondary use of collected data was obtained from the Health Sciences and Behavioral Sciences (HSBS) IRB at the University of Michigan (HUM00264428). Ethical approval for data collection in West Texas was obtained in May 2025 from the University of Michigan HSBS IRB, with further approval by University of Michigan Medical School IRB (HUM00263619). Electronic written informed consent for participation was obtained from all subjects in-volved in both studies. Copies of informed consent forms for each study are included in the Supplementary Material.

### Study Population and Recruitment

This study was nested within two larger cross-sectional serological and skin test studies of employees at Kern Medical (KM) in Bakersfield, California and members of the general public within several counties in the Permian Basin region of West Texas. The study populations were chosen to capture a range of awareness of Valley fever within regions where Valley fever is believed to be endemic. For each study population, we administered a survey to gather information on participant knowledge of Valley fever, history of coccidioidomycosis infection or pneumonia-like illness, demographic and behavioral risk factors for infection, and willingness to receive a human vaccine and, for those who had dogs, willingness to vaccinate their dogs. In the same survey, participants were then asked if they would be willing to answer additional questions related to factors influencing their willingness to receive a future Valley fever vaccine. Target sample sizes for the study were pre-determined based on estimating prevalence of prior infection via skin or serological test result.

Participants at KM were recruited through volunteer sampling of the employee population from March to September 2025, inclusive of both healthcare workers and non-healthcare workers. Employees interested in contributing to the broader Valley fever serological and skin test study filled out an interest form and were contacted for recruitment. The interest form was distributed by email to all employees, advertised on employees’ work computers, and posted in common spaces at KM (e.g., elevators, cafeterias). The interest form closed at 150 responses, with the target of enrolling 100 participants. Participants who could commit to the study timeline, were 18 years of age or older, and who were not currently pregnant were eligible for the study. After obtaining informed consent, eligible participants were enrolled in the study and completed the questionnaire.

In the West Texas population, participants were also recruited for a broader serological study using volunteer sampling in the endemic counties of Midland, Ector, Martin, and Howard from August to October of 2025. Utilizing partnerships established with Texas Tech University, Texas Tech University Health Sciences Center—Clinical Research Institute, local organizations and religious groups, we conducted 10 pop-up booths situated at community events and local health clinics to recruit and enroll participants. A recruitment poster was displayed and flyers were distributed to potential participants at these locations, and the survey was completed on-site after obtaining informed consent. Recruitment materials, informed consent forms, and the survey were available in both English and Spanish. Individuals who were 18 years of age or older, not knowingly pregnant, absent a diagnosis of sickle cell disease or anemia, and without cold or flu symptoms at the time of enrollment were eligible for participation.

### Questionnaire

The survey administered in both study populations was adapted from the California Department of Public Health’s 2023 Enhanced Surveillance questionnaire administered to patients with confirmed coccidioidomycosis diagnoses [24] and from the 2008 Behavioral Risk Factor Surveillance System questionnaire used in Arizona, which contained state-added questions on Valley fever [25,26]. We developed and administered this survey and stored survey responses in REDCap, an electronic data capture tool hosted at University of Michigan (Ann Arbor, MI, USA). The survey captured information from consented participants on demographics, immune status, knowledge of Valley fever, history of Valley fever-like illness, symptoms, and prior diagnosis, as well as engagement in known risk factors for Valley fever. Participants were also asked whether they would be willing to receive a future Valley fever vaccine, whether they would vaccinate their dogs with a Valley fever vaccine, and the degree to which characteristics and circumstances surrounding Valley fever vaccination, including vaccine side effects or easy access to a vaccination clinic, would influence their willingness to self-vaccinate. A copy of this questionnaire is included in the Supplementary Material.

### Variables

Two primary outcomes of interest were defined: (1) willingness to consider receiving a Valley fever vaccine in the future, should one become available, and (2) among individuals with pet dogs, willingness to vaccinate their dogs against Valley fever, should a vaccine become available. For each endpoint, participants could answer “Yes”, “No”, or “Don’t know/Unsure”; these levels were retained for the descriptive analysis and levels of “No” and “Don’t know/Unsure” were combined into a single reference group for regression analyses. Vaccine willingness was descriptively assessed across demographic categories and risk factors for Valley fever. Demographic categories included age group (defined as 18–30, 31–40, 41–50, 51–65, and 66+ years), sex (male or female), and race/ethnicity (combined categories of Hispanic, Non-Hispanic White, and Non-Hispanic Other). Immunocompromised status was assessed as a binary variable.

Behavioral, occupational, and lifestyle risk factors for Valley fever included frequency of exposure to soil (through digging in or disturbing soil for work or recreation), frequency of exposure to dust through outdoor leisure activities, exposure to dust storms, history of an outdoor occupation (including wildland firefighting, construction, agriculture, archaeology, military, mining, gas and oil extraction, and other occupations), and previous knowledge of Valley fever. Frequency of exposure to soil and dust were each measured on a five-point Likert scale, including “Almost every day”, “A few days a week”, “A few times a month”, “A few times a year”, and “Never”. For analysis, these levels were dichotomized into “More than once a month” and “Less than once a month”.

Additional variables measured participant willingness to receive a Valley fever vaccine on a four-point Likert scale from “Much less likely” to “Much more likely” under ten conditions: (1) the vaccine were free, (2) a vaccine clinic was easily accessible, or (3) there was free transportation to it, (4) the vaccine had no side effects, or (5) its side effects affected daily tasks, (6) they were able to take off work/school to deal with side effects, (7) it was doctor or healthcare provider recommended, (8) a family member or friend received it, (9) a religious leader or community leader they trust received it, and (10) they would be able to safely engage in outdoor recreational activities if they received it. A “Did not affect decision” option was available for these questions in the Bakersfield survey population.

### Calculation of Survey Weights

We calculated survey weights to increase comparability between the sampled populations and the underlying Bakersfield and West Texas populations. Using data from the American Community Survey’s Public Use Microdata Sample (PUMS) [27], we determined the joint probability of belonging to each combination of age group (18–30, 31–40, 41–50, 51–65, 66+), sex (male or female), and race/ethnicity (Hispanic, Non-Hispanic White, Non-Hispanic Black, Non-Hispanic Asian/Pacific Islander, and Non-Hispanic Other Race) within Bakersfield, California and the Permian Basin region of Texas. We then determined the joint probability of belonging to the same categories among the study samples. As age was not a required question in the West Texas sample, we imputed occasional missing values for age based on reported length of residence in Texas, other states, and in the U.S. broadly. Observations with missing age and incomplete residence history were dropped from the analysis. We computed post-stratification weights by dividing the PUMS microdata proportions by their respective study sample proportions. We truncated weights for both samples at the 2.5th and 97.5th percentiles to reduce any over- or under-representation of joint distribution groups.

### Statistical Analysis

Descriptive analyses were performed to compare willingness to receive a Valley fever vaccine across demographic and risk factor categories and survey location (Bakersfield, CA or West TX). Willingness to vaccinate pet dogs was assessed by participant demographic factors only. Unweighted and survey-weighted proportions were calculated, and Wald 95% confidence intervals (CIs) were computed. Given that most employees at KM were anticipated to have knowledge about Valley fever, we only considered awareness of Valley fever as a comparison group for the West Texas sample. Tables with the weighted results are included in the main text, and unweighted results are included in the Supplementary Material.

Quasi-binomial logistic regression models were fitted to examine unadjusted, bivariate associations between demographic characteristics and risk factors for Valley fever and survey participants’ willingness to vaccinate themselves and their dogs. Regression models for Valley fever risk factors were further adjusted for age, sex, and race/ethnicity. We reported the crude and adjusted odds ratios and 95% CIs for the association between each demographic and risk factor and willingness to receive a Valley fever vaccine.

Further descriptive analyses were performed among the subset of participants who answered additional survey questions about factors that would influence their decision to receive a Valley fever vaccine. Because the factors influencing vaccine uptake may be of greater concern to participants who are unwilling to receive a vaccine or unsure about receiving a vaccine, as compared to participants already willing to receive a vaccine, these analyses were split into two strata: participants who expressed uncertainty/unwillingness to vaccinate themselves and participants who expressed willingness to vaccinate themselves against Valley fever. For each factor potentially influencing vaccine willingness, we computed survey-weighted percentages for each Likert scale category, indicating the conditions under which individuals would be more or less likely to receive a Valley fever vaccine in Bakersfield, CA and West Texas.

Statistical analyses were conducted in R (versions 4.5.3 and 4.5.2) using the “survey” package to account for survey weights [28,29]. Generative artificial intelligence (GenAI) was not used to generate text, data, or graphics, or to assist in study design, data collection, analysis, or interpretation.

## 3. Results

We enrolled 104 participants at Kern Medical (KM) in Bakersfield, CA, with 103 participants who completed the questionnaire. In West Texas, we enrolled 245 participants. After excluding individuals with missingness in age as well as an individual who responded twice, we had 230 participants eligible for inclusion in this analysis. Among KM employees, the majority were female (79.6%) and 18–50 years of age (76.7%), with 46 (44.7%) identifying as Hispanic, 38 (36.9%) identifying as Non-Hispanic White, and 19 (18.4%) identifying as a Non-Hispanic Other race (i.e., Non-Hispanic Black, Non-Hispanic Asian/Pacific Islander, Non-Hispanic Other, or Mixed Race). Among the West Texas population, which included respondents from sixteen counties, the majority were female (68.7%), with relatively equal age distribution (18.7% to 21.3% per age group). The majority of participants from West Texas identified as Hispanic (56.5%), or Non-Hispanic White (35.7%), and the remaining 7.8% were of Non-Hispanic Other race (Supplementary Table S1).

### 3.1. Intent to Receive a Valley Fever Vaccine

An estimated three-fourths of KM employees expressed willingness to vaccinate themselves with a Valley fever vaccine (weighted percentage: 76%, 95% CI: 63–85%), while 20% (95% CI: 12–34%) were unsure, and 4% (95% CI: 2–9%) were unwilling to self-vaccinate (Table 1; unweighted percentages in Supplementary Table S2). A higher proportion of males (86%, 95% CI: 54–97%) were willing to receive a Valley fever vaccine compared to females (69%, 95% CI: 55–81%). Willingness to self-vaccinate was generally high within each age group, but uncertainty about vaccination was highest among those aged 41–50 years (29%, 95% CI: 12–56%). Uncertainty about vaccination was more common among participants who identified as a Non-Hispanic race other than White (36%, 95% CI: 14–66%) compared to those who were Non-Hispanic White (11%, 95% CI: 4–27%) or Hispanic (23%, 95% CI: 10–45%). Willingness to vaccinate was also lower among those who were immunocompromised compared to those who were not immunocompromised (68%, 95% CI: 30–91% versus 77%, 95% CI: 64–87%).

Among KM employees in Bakersfield, those who reported digging in or disturbing the soil more than once per month were more willing to obtain a vaccine than those who dug in or disturbed soil less often or never (85%, 95% CI: 60–96% versus 73%, 95% CI: 58–84%). High willingness to vaccinate was also observed among those who participated in leisure activities that exposed them to dust more than once a month (73%, 95% CI: 56–85%) and those who ever worked in an outdoor occupation (92%, 95%CI: 67–98%). However, only 65% (95% CI: 47–80%) of those who had been caught in a dust storm were willing to receive a Valley fever vaccine while 95% (95% CI: 85–98%) of those who had not been caught in a dust storm were willing (Table 1).

Among the general public in West Texas, there was a higher degree of uncertainty surrounding participants’ willingness to vaccinate themselves against Valley fever. Overall, an estimated 42% (95% CI: 34–49%) of the West Texas population reported being willing to receive the vaccine, 42% (95% CI: 34– 49%) were unsure if they would receive the vaccine, and 17% (95% CI: 12–23%) were unwilling (Table 1). Vaccine willingness did not meaningfully differ by sex. Willingness to self-vaccinate followed a decreasing trend by age for those under 65 years old, with 51–65-year-old participants being most unwilling (28%, 95% CI: 15–47%). Non-Hispanic White individuals were the least willing to receive a Valley fever vaccine (34%, 95% CI: 24–47%) compared to Hispanic (47%, 95% CI: 36–57%) and Non-Hispanic Other race (49%, 95% CI: 22–76%) individuals. Participants who were not immunocompromised were more willing to accept the vaccine compared to those who were immunocompromised (44%, 95% CI: 35–52% versus 32%, 95% CI: 20–49%), with more than half of all those who were immunocompromised uncertain about vaccination (51%, 95% CI: 35–67%).

Among the West Texas population, individuals who were previously aware of Valley fever were more willing to receive a vaccine than those who were unaware of Valley fever (64%, 95% CI: 46–79% versus 37%, 95% CI: 29–45%). Those who dug in or disturbed the soil more than once a month were more uncertain about vaccination (47%, 95% CI: 34–61%) than those who dug or disturbed the soil less than once a month or never (39%, 95% CI: 30–48%). Vaccine willingness was lower for those who participated in leisure activities that exposed them to dust more than once a month in comparison to less than once a month or never (39%, 95% CI: 30–48% versus 47%, 95%CI: 33–61%). For individuals who had been caught in a dust storm, willingness to vaccinate themselves was lower than for individuals who had not been caught in a dust storm (40%, 95% CI: 31–49% versus 47%, 95% CI: 31–63%). Individuals who had ever had an outdoor occupation were less willing (37%, 95% CI: 24–53%) to receive a Valley fever vaccine than those with indoor occupations (43%, 95% CI: 34–51%) (Table 1).

### 3.2. Owner Willingness to Vaccinate Their Dog Against Valley Fever

Of individuals at KM who owned a dog (n = 78), an estimated 74% (95% CI: 58–86%) were willing to vaccinate their dog against Valley fever, while 25% (95% CI: 14–41%) were unsure and 1% (95% CI: 0–6%) were unwilling (Table 2; unweighted percentages in Supplementary Table S3). Females were more willing to vaccinate their dog (79%, 95% CI: 66–88%) than were males (66%, 95% CI: 31–90%). In each age group, most were willing to vaccinate their dog, but willingness was lowest among those aged 18–30 (61%, 95% CI: 27–86%). Uncertainty toward vaccinating dogs was highest among those who identified as a Non-Hispanic race other than White (34%, 95% CI: 9–72%) (Table 2).

Among dog owners in West Texas (n = 178), an estimated 49% (95% CI: 41–58%) were willing to vaccinate their dog, 35% (95% CI: 27–44%) were unsure and 16% (95% CI: 10–23%) were not willing to vaccinate their dog (Table 2). Males were slightly more unsure than females (37%, 95% CI: 24–52% versus 33%, 95% CI: 25–43%). Individuals aged 41–50 were the most unwilling to vaccinate their dogs (24%, 95% CI: 10–49%), and individuals aged 51–65 were the most uncertain about vaccinating their dogs (39%, 95% CI: 24–57%). In comparison to other racial/ethnic groups, Non-Hispanic White individuals were the most unsure about vaccinating their dogs (45%, 95% CI: 33–57%) (Table 2).

### 3.3. Factors Associated with Intent to Receive a Valley Fever Vaccine

Immunocompromised status, digging in or disturbing soil, leisure activities with dust exposure, and ever having an outdoor occupation did not show statistically significant associations with vaccine willingness in either study location (Table 3). In Bakersfield, CA, ever being caught in a dust storm showed an inverse association with willingness to receive a Valley fever vaccine, adjusting for age, sex, and race/ethnicity (adjusted odds ratio (aOR): 0.10, 95% confidence interval (CI): 0.02–0.40); this association was not statistically significant in West Texas. In West Texas, those with prior awareness of Valley fever had 3.83 (95% CI: 1.73–8.49) times the odds of being willing to receive a Valley fever vaccine compared to those who had never heard of Valley fever, after adjusting for age, sex, and race/ethnicity.

### 3.4. Factors Increasing Intent to Vaccinate Against Valley Fever

Among participants who responded to the questionnaire, 75 in the Bakersfield study and 104 in the West Texas study answered optional questions on the degree to which certain conditions would influence their willingness to receive a Valley fever vaccine. Of the 75 respondents in Bakersfield, 57 (estimated weighted percentage: 76%) were willing to receive the vaccine and 18 (24%) were unsure or unwilling, and of the 104 respondents in West Texas, 58 (56%) were willing to receive the vaccine, and 46 (44%) were unsure or unwilling.

**Figure 1:**
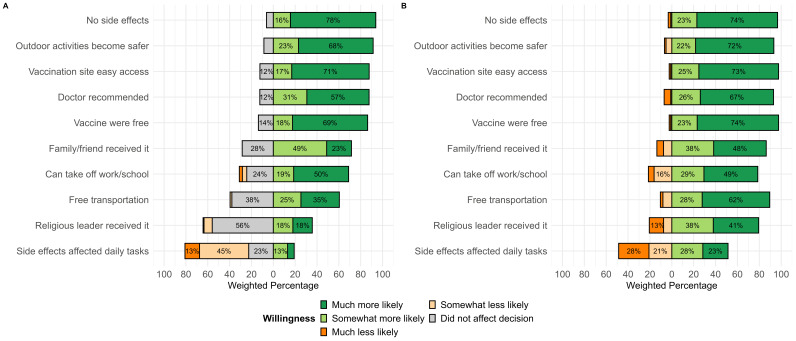
Among those who were willing to self-vaccinate and answered additional vaccine willingness questions, factors influencing willingness to receive a Valley fever vaccine in (**A**) Bakersfield, California (N = 57) and (**B**) West Texas (N = 58).

**Figure 2:**
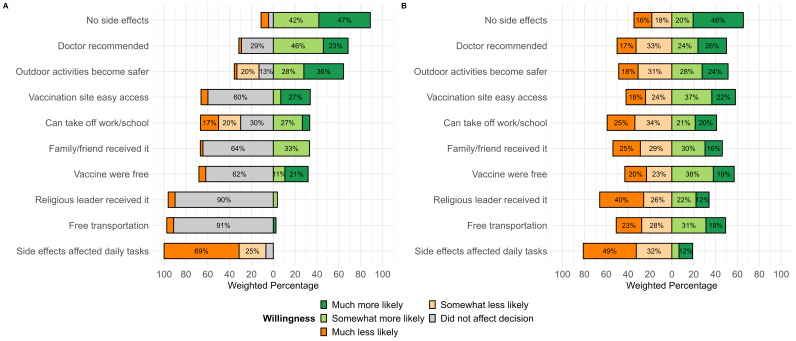
Among those who were uncertain or unwilling to self-vaccinate and answered additional vaccine willingness questions, factors influencing willingness to receive a Valley fever vaccine in (**A**) Bakersfield, California (N = 18) and (**B**) West Texas (N = 46).

Among both study populations, a vaccine without any side effects was identified as the most influential factor that would increase willingness to vaccinate. In the Bakersfield population, an estimated 78% of those who were willing to vaccinate and 47% of those who were unsure about or unwilling to vaccinate reported that they would be “much more likely” to vaccinate if this condition were met (Figure 1A and Figure 2A). Similarly, in the West Texas population, 74% of those who were willing to vaccinate and 46% of those who were unsure or unwilling reported that they would be “much more likely” to vaccinate if there were no vaccine side effects (Figure 1B and Figure 2B). Similarly, in both populations, the most inhibiting factor to vaccination was the possibility of the vaccine’s side effects affecting daily activities; an estimated 13–28% of those willing to be vaccinated reported that they would be “much less likely” to vaccinate given vaccine side effects, while an estimated 49–69% of those unsure or unwilling also indicated they would also be “much less likely” to vaccinate themselves if this condition were true. Among those willing and unwilling to vaccinate in either study site, other factors that would increase the likelihood that respondents would be willing to receive the Valley fever vaccine included if the vaccine was doctor recommended, made outdoor activities safer, was free, and was available at an accessible vaccination site.

## 4. Discussion

As the burden of Valley fever continues to increase across the American Southwest, generating a well-tolerated, effective coccidioidal vaccine and gaining broad vaccine acceptance among the public remain some of the biggest hurdles to introducing a vaccine aimed at reducing Valley fever morbidity. In this study, we examined the willingness to accept human and canine vaccines against Valley fever among two geographically different study populations spanning a wide range of awareness of Valley fever. We found that willingness to vaccinate against symptomatic coccidioidomycosis varied by study population, with employees of Kern Medical (KM) in Bakersfield, CA—where awareness of Valley fever is high—reporting a relatively high level of willingness to vaccinate themselves (76%) and, among dog owners, their canine companions (74%). Self-vaccination willingness was much lower among members of the general population in West Texas (42%) as was willingness to vaccinate their dogs (49%). We found that the most important predictor of vaccination among the West Texas population was prior awareness of Valley fever, with 64% of those with prior awareness being willing to vaccinate, compared with only 37% of those without prior awareness. Other factors increasing willingness to receive a future Valley fever vaccine were similar in each study population and among those certain and uncertain about self-vaccination. Overall, the absence of vaccination side effects was the most encouraging factor, while the potential that side effects would affect daily activities was the most dissuading.

Our results suggest that interest in a Valley fever vaccine is comparable to that of other one-time vaccines and may exceed that of seasonal vaccines. For instance, in comparison to the seasonal influenza vaccine, intent to accept a Valley fever vaccine was higher than influenza acceptance rates. By the end of the 2024–2025 influenza season, statewide vaccination in California was 30.4% for adults, with a higher percentage among adults older than 65 [30]. In Texas, the statewide overall adult vaccination rate by the same date was 10.2% and was 23.9% for adults older than 65 [30]. In our study, of the 17% of West Texas participants who previously knew of Valley fever, 64% reported willingness to vaccinate, while of the 83% who were previously unaware, 37% reported willingness to vaccinate; both willingness levels are higher than the state’s seasonal influenza uptake rate among adults in 2025. Willingness to receive a Valley fever vaccine is also comparable to current uptake of the one-time, recommended measles, mumps, and rubella (MMR) vaccine. In Kern County (Bakersfield) and Midland and Ector County (two of the most populous counties in West Texas), the MMR vaccination rates for children under 5 in 2025 were between 57% and 65% [31], sitting between the vaccine willingness levels observed in West Texas and Bakersfield.

Our results also suggest that increased knowledge or awareness of a disease may influence vaccine willingness, even in vaccine-hesitant regions. In West Texas, those who indicated that they were previously aware of Valley fever were almost twice as willing to receive the vaccine, while employees of Kern Medical—who are assumed to have at least some disease awareness—also had high willingness to accept the vaccine. Knowledge of disease circulation may be important for increasing uptake of other vaccines as well. One study found that within a month of the 2025 West Texas measles outbreak, there was a significant spike in early MMR vaccination among children [32]. What is more, while tetanus is extremely rare in the United States, the name and dangers associated with the disease are well-known amongst the general population [33]. In 2013, the national average vaccination coverage among adults for either Tdap or Td vaccines was 86.4%, and in California and Texas, adult coverage exceeded 88% and 82%, respectively [34]. High vaccination rates against tetanus suggest that for an environmentally-acquired rare disease—much like Valley fever—global recognition of the disease may bolster vaccination rates, even among individuals who might be hesitant to receive childhood vaccinations or seasonal influenza vaccinations. In Texas, where Valley fever is not reportable outside of El Paso County, state recognition of Valley fever disease severity and its risk factors may drive up willingness to receive a future vaccine, even as we found that acceptance was not universal among people who were aware of the disease. Beyond awareness and low side effects, participants in this study also indicated doctor recommendations as a key factor increasing willingness. Previous work on COVID-19 vaccine acceptance found high trust in healthcare workers [35], and when equipped with proper tools and training, especially in motivational interviewing, healthcare workers can further tease apart and intervene on reasons for hesitancy [36]. However, while intervening on modifiable factors like education through public health campaigns might increase willingness and vaccination coverage, the current climate of vaccination hesitancy is the result of many influences, including strong political and social pressures unmeasured in this study.

Contrary to expectations, in both studies, participants who spent more time engaging in activities that exposed them to outdoor dust—which may co-occur with *Coccidioides* spp. Spores—were less willing to vaccinate themselves than those who spent less or no time; however, this factor was not statistically significant in adjusted logistic regression models and therefore could have reflected differences in demographic composition of those who spend more time engaged in dust-generating activities. It is also possible that individuals who regularly participate in activities that expose them to dust do not perceive such activities as high risk, even as exposure to outdoor dust aerosolized from spore-containing soils, such as undisturbed soils of undeveloped land [37], has been found to be positively associated with coccidioidomycosis disease onset 1–3 months later [38]. We also found that there was a high level of uncertainty toward vaccination in those who had ever been caught in a dust storm: 31% in Bakersfield and 46% in West Texas. In Bakersfield, those who had ever been caught in a dust storm had statistically significantly lower odds of being willing to vaccinate than those who had never been in a dust storm. Because knowledge of Valley fever and its risk factors was high among the Bakersfield sample, this could have led those who were previously in a dust storm to believe they had been previously infected and therefore perceive the vaccine as not necessary.

This study has limitations. Both study sample populations were gathered using volunteer sampling, which may introduce selection bias through selecting participants who are more health conscious and who may be both more motivated to participate in health studies and more willing to receive vaccines [39]. Similarly, because this study was nested within a broader serologic study in each study site, this analysis could not determine vaccination willingness in those with exclusionary conditions such as sickle cell anemia or pregnancy, or among those unwilling to undergo fingerpoke blood collection or a *Coccidioides* skin test. This may further bias our estimates of vaccine willingness upward as individuals unwilling to undergo fingerpoke blood collection or skin testing may be less willing to undergo vaccination. At the same time, vaccination hesitancy in pregnant individuals or those with exclusionary medical conditions may be more dynamic which could further bias our estimates in either direction [40]. Second, the populations sampled may not be generalizable to other populations. This is especially true of the employee population of Kern Medical, where many individuals were healthcare professionals and knowledge of Valley fever should be universal. Although we employed post-stratification weighting to correct for oversampling and undersampling of the source population by age, sex, and race/ethnicity demographics to reduce selection bias and increase generalizability, the proportion willing to vaccinate may still be lower in the general population.

Sample sizes for this study were based on sample sizes for the larger serological or skin test surveys. As a result, this study is limited by the relatively small sample size for both study populations, resulting in wide 95% confidence intervals which may hinder our ability to draw definitive conclusions about reported willingness to be vaccinated and factors influencing vaccination. Questionnaire responses are additionally subject to information bias. Within the West Texas sample, imputation of some missing age data based on residence history may have resulted in some age misclassification. However, age groupings and post-stratification weighting may correct for minor discrepancies between imputed age and true age. Further, question misinterpretation and/or survey fatigue may have also affected the reliability of responses. For example, some West Texas participants indicated in the final set of survey questions that the vaccine having side effects would make them somewhat or much more likely to be vaccinated, when one would expect the opposite; either question misinterpretation or survey fatigue may explain this discrepancy. Lastly, the outcome question on intent to receive a future Valley fever vaccine for oneself or for their dog did not describe the protection offered by the vaccine, whether its primary outcome is to generate sterilizing immunity or reduce disease severity. As such, we can only claim the observed willingness levels among participants reflect a generic understanding of Valley fever vaccination; true vaccine acceptance may vary by vaccine efficacy and the type of protection offered. Additionally, vaccination willingness is likely to overestimate vaccination coverage as other factors unexplored in this study may limit access and motivations to vaccinate. Upon the release of a commercially available vaccine for Valley fever, future study should examine factors that prevent an otherwise willing individual from becoming vaccinated.

Despite limitations, our study contributes new insight into individual willingness to receive a human or canine coccidioidomycosis vaccine and the factors that lessen vaccine hesitancy in humans. A key strength of this study is its application in two diverse populations. Reaching participants with varying degrees of Valley fever disease awareness facilitated investigation into the possible range of vaccination willingness and reasons for vaccine hesitancy. Prior to this study, vaccination willingness in areas such as West Texas, where the disease is endemic but not reportable and which is a known canine coccidioidomycosis hotspot, was relatively unknown. Our results also offer several implications for the planning and rollout of a future Valley fever vaccine in the United States. Prior to vaccine rollout, Valley fever awareness campaigns should be expanded with messaging tailored to cohorts who reported lower willingness to be vaccinated: those who frequently dig in or disturb the soil, are frequently exposed to dust in work, recreation, or leisure activities, and who may be exposed to dust storms.

## 5. Conclusions

As *Coccidioides* spp. expands into new geographic regions, increasing the number of people at-risk for exposure to the fungus, determining priority groups for vaccination and factors that may influence vaccination willingness may help to reduce disease incidence and severity. Our findings suggest that there would be substantial demand for a Valley fever vaccine in both humans and dogs, further motivating the goal to send coccidioidal vaccine candidates to human trials by 2030. Nevertheless, our results also suggest that, to reduce vaccine hesitancy, increasing awareness of Valley fever in the targeted population and prioritizing vaccine candidates with few adverse effects should be prioritized.

## Figures and Tables

**Table 1:** Willingness to vaccinate self against Valley fever by demographic and risk factors for coccidioidomycosis.

	Bakersfield, CA (N = 103)				West TX (N = 230)			
		Weighted Percentage, % (95% CI)				Weighted Percentage, % (95% CI)		
	n	Willing	Unsure	No	n	Willing	Unsure	No
**Overall**	103	76 (63, 85)	20 (12, 34)	4 (2, 9)	230	42 (34, 49)	42 (34, 49)	17 (12, 23)
**Sex**								
Male	21	86 (54, 97)	13 (3, 46)	1 (0, 11)	72	43 (30, 56)	42 (30, 55)	15 (8, 27)
Female	82	69 (55, 81)	25 (14, 40)	6 (2, 13)	158	41 (33, 49)	41 (33, 50)	18 (12, 26)
**Age (years)**								
18–30	26	78 (47, 93)	20 (5, 52)	2 (0, 17)	43	52 (34, 69)	36 (21, 55)	12 (5, 27)
31–40	29	79 (60, 91)	8 (3, 23)	12 (4, 30)	49	42 (28, 58)	47 (33, 62)	10 (4, 24)
41–50	24	66 (41, 85)	29 (12, 56)	4 (1, 27)	45	37 (22, 56)	46 (29, 64)	17 (7, 34)
51–65	19	74 (47, 90)	26 (10, 53)	0	49	25 (14, 40)	46 (31, 62)	28 (15, 47)
66+	5	76 (12, 99)	24 (1, 88)	0	44	54 (36, 71)	29 (15, 47)	17 (8, 35)
**Race/Ethnicity**								
Hispanic	46	73 (52, 87)	23 (10, 45)	4 (1, 12)	130	47 (36, 57)	36 (27, 47)	17 (11, 27)
Non-Hispanic White	38	83 (67, 92)	11 (4, 27)	5 (2, 15)	82	34 (24, 47)	49 (38, 61)	16 (9, 26)
Non-Hispanic Other	19	64 (34, 86)	36 (14, 66)	0	18	49 (22, 76)	35 (14, 64)	17 (2, 64)
**Immunocompromised status**								
Immunocompromised	18	68 (30, 91)	29 (7, 69)	2 (0, 18)	48	32 (20, 49)	51 (35, 67)	16 (8, 32)
Not immunocompromised	85	77 (64, 87)	18 (10, 32)	4 (2, 10)	182	44 (35, 52)	40 (32, 48)	17 (11, 24)
**Frequency of digging in soil**								
More than once a month	19	85 (60, 96)	13 (3, 39)	2 (0, 17)	74	34 (23, 48)	47 (34, 61)	19 (10, 31)
Less than once a month	84	73 (58, 84)	22 (12, 38)	4 (2, 10)	154	45 (36, 54)	39 (30, 48)	16 (10, 24)
Missing	0	-	-	-	2	-	-	-
**Frequency of leisure activities that expose to dust**								
More than once a month	76	73 (56, 85)	23 (11, 40)	5 (2, 11)	159	39 (30, 48)	45 (36, 54)	16 (11, 24)
Less than once a month	27	84 (65, 94)	14 (5, 34)	2 (0, 13)	67	47 (33, 61)	35 (23, 49)	18 (10, 32)
Missing	0	-	-	-	4	-	-	-
**Ever caught in a dust storm**								
Yes	63	65 (47, 80)	31 (17, 50)	4 (1, 11)	171	40 (31, 49)	46 (37, 54)	15 (10, 22)
No	32	95 (85, 98)	2 (0, 10)	3 (1, 13)	46	47 (31, 63)	27 (15, 43)	27 (14, 45)
Unsure	8	57 (18, 89)	33 (7, 77)	9 (1, 57)	9	50 (12, 88)	47 (11, 87)	4 (0, 32)
Missing	0	-	-	-	4	-	-	-
**Ever had an outdoor occupation**								
Yes	17	92 (67, 98)	8 (2, 33)	0	56	37 (24, 53)	51 (36, 66)	12 (6, 24)
No	86	71 (56, 82)	24 (13, 40)	5 (2, 11)	172	43 (34, 51)	38 (30, 47)	19 (13, 28)
Missing	0	-	-	-	2	-	-	-
**Previous awareness of Valley fever**								
Previously aware					40	64 (46, 79)	28 (15, 46)	8 (3, 21)
Previously unaware					190	37 (29, 45)	45 (36, 53)	19 (13, 26)

**Table 2:** Willingness to vaccinate dogs against Valley fever by participant demographics.

	Bakersfield, CA (N = 78)				West TX (N = 178)			
		Weighted Percentage, % (95% CI)				Weighted Percentage, % (95% CI)		
	n	Willing	Unsure	No	n	Willing	Unsure	No
**Overall**	78	74 (58, 86)	25 (14, 41)	1 (0, 6)	178	49 (41, 58)	35 (27, 44)	16 (10, 23)
**Sex**								
Male	15	66 (31, 90)	34 (10, 69)	0	55	48 (34, 62)	37 (24, 52)	15 (7, 29)
Female	63	79 (66, 88)	20 (11, 32)	1 (0, 10)	123	51 (41, 61)	33 (25, 43)	16 (10, 25)
**Age**								
18–30	18	61 (27, 86)	39 (14, 73)	0	32	52 (31, 72)	36 (18, 59)	12 (4, 28)
31–40	24	75 (48, 91)	21 (7, 49)	4 (0, 25)	42	53 (36, 68)	35 (21, 52)	12 (5, 28)
41–50	22	73 (46, 89)	27 (11, 54)	0	34	48 (29, 68)	27 (13, 49)	24 (10, 49)
51–65	10	76 (41, 94)	24 (6, 59)	0	42	42 (26, 59)	39 (24, 57)	19 (9, 38)
66+	4	100	0	0	28	55 (33, 75)	35 (17, 58)	10 (3, 30)
**Race/Ethnicity**								
Hispanic	37	72 (48, 88)	26 (11, 50)	1 (0, 11)	90	52 (39, 64)	27 (17, 40)	21 (13, 33)
Non-Hispanic White	31	79 (57, 92)	21 (8, 43)	0	77	47 (35, 59)	45 (33, 57)	8 (4, 17)
Non-Hispanic Other	10	66 (28, 91)	34 (9, 72)	0	11	52 (17, 85)	17 (3, 57)	31 (5, 77)

**Table 3:** Unadjusted and adjusted * association of risk factors with willingness to vaccinate self.

	Bakersfield, CA				West TX			
	Unadjusted Association		Adjusted Association		Unadjusted Association		Adjusted Association	
	OR (95% CI)	*p*-Value	OR (95% CI)	*p*-Value	OR (95% CI)	*p*-Value	OR (95% CI)	*p*-Value
**Immunocompromised status**								
Immunocompromised (ref: not immunocompromised)	0.63 (0.12, 3.22)	0.57	0.73 (0.18, 2.97)	0.66	0.62 (0.29, 1.32)	0.21	0.59 (0.26, 1.33)	0.20
**Frequency of digging in the soil**								
More than once a month (ref: less than once a month)	2.05 (0.49, 8.61)	0.32	2.38 (0.61, 9.33)	0.21	0.64 (0.32, 1.28)	0.21	0.62 (0.29, 1.33)	0.22
**Frequency of leisure activities that expose to dust**								
More than once a month (ref: less than once a month)	0.50 (0.14, 1.76)	0.28	0.37 (0.1, 1.36)	0.14	0.71 (0.36, 1.41)	0.33	0.79 (0.39, 1.57)	0.49
**Ever caught in a dust storm**								
Yes (ref: no)	0.10 (0.03, 0.41)	**<0.01**	0.10 (0.02, 0.4)	**<0.01**	0.73 (0.35, 1.52)	0.40	0.84 (0.40, 1.79)	0.65
**Ever had an outdoor occupation**								
Yes (ref: no)	4.50 (0.81, 25.12)	0.09	6.09 (0.89, 41.68)	0.07	0.78 (0.38, 1.61)	0.51	0.71 (0.28, 1.83)	0.48
**Previous awareness of Valley fever**								
Previously aware (ref: previously unaware or unsure)					3.09 (1.39, 6.84)	**<0.01**	3.83 (1.73, 8.49)	**<0.01**

* Adjusted for age, sex, and race/ethnicity of respondent. OR: odds ratio.

## Data Availability

The de-identified data supporting the conclusions of this article will be made available upon request from the corresponding author.

## Supplementary Materials

The following supporting information can be downloaded at: https://www.mdpi.com/article/10.3390/jof12060420/s1: Supplementary Table S1: Participant demographics in the Bakersfield and West Texas survey populations, Supplementary Table S2: Unweighted crosstabulations of participant willingness to vaccinate self by coccidioidomycosis risk factor, Supplementary Table S3: Unweighted crosstabulations of participant willingness to vaccinate their dog by coccidioidomycosis risk factor, a copy of the study questionnaire, and copies of the consent forms used at Kern Medical and in West Texas.

## Abbreviations

The following abbreviations are used in this manuscript:

OR	Odds ratio
aOR	Adjusted odds ratio
CI	Confidence interval
KM	Kern Medical
PUMS	Public Use Microdata Sample

## References

[1] Galgiani J.N., Ampel N.M., Blair J.E., Catanzaro A., Johnson R.H., Stevens D.A., Williams P.L., Infectious Diseases Society of America (2005). Coccidioidomycosis. Clin. Infect. Dis..

[2] Laniado-Laborin R. (2007). Expanding Understanding of Epidemiology of Coccidioidomycosis in the Western Hemisphere. Ann. N. Y. Acad. Sci..

[3] Donovan F.M., Ampel N.M., Thompson G.R. (2025). Coccidioidomycosis. Infect. Dis. Clin. N. Am..

[4] McCotter O.Z., Benedict K., Engelthaler D.M., Komatsu K., Lucas K.D., Mohle-Boetani J.C., Oltean H., Vugia D., Chiller T.M., Sondermeyer Cooksey G.L. (2019). Update on the Epidemiology of coccidioidomycosis in the United States. Med. Mycol..

[5] Williams S.L., Benedict K., Jackson B.R., Rajeev M., Cooksey G., Ruberto I., Williamson T., Sunenshine R.H., Osborn B., Oltean H.N. (2025). Estimated Burden of Coccidioidomycosis. JAMA Netw. Open.

[6] Gorris M.E., Ardon-Dryer K., Campuzano A., Castañón-Olivares L.R., Gill T.E., Greene A., Hung C.-Y., Kaufeld K.A., Lacy M., Sánchez-Paredes E. (2023). Advocating for Coccidioidomycosis to Be a Reportable Disease Nationwide in the United States and Encouraging Disease Surveillance across North and South America. J. Fungi.

[7] Benedict K., McCotter O.Z., Brady S., Komatsu K., Sondermeyer G.L., Cooksey, Nguyen A., Jain S., Vugia D.J., Jackson B.R. (2019). Surveillance for Coccidioidomycosis—United States, 2011–2017. MMWR Surveill. Summ..

[8] Fan H., Donovan F., Lovelace B., Coleman C.I. (2025). Coccidioidomycosis-Attributable Death in the United States: An Analysis of Cases Reported on Death Certificates, 2018–2023. J. Fungi.

[9] Grizzle A.J., Wilson L., Nix D.E., Galgiani J.N. (2021). Clinical and Economic Burden of Valley Fever in Arizona: An Incidence-Based Cost-of-Illness Analysis. Open Forum Infect. Dis..

[10] Shubitz L.F., Butkiewicz C.D., Dial S.M., Lindan C.P. (2005). Incidence of Coccidioides infection among dogs residing in a region in which the organism is endemic. J. Am. Vet. Med. Assoc..

[11] Teel K.W., Yow M.D., Williams T.W. (1970). A localized outbreak of coccidioidomycosis in southern Texas. J. Pediatr..

[12] Sykes J.E., Camponuri S.K., Weaver A.K., Thompson G.R., Remais J.V. (2025). Use of dog serologic data for improved understanding of coccidioidomycosis: A One Health approach. J. Infect. Dis..

[13] Gorris M.E., Treseder K.K., Zender C.S., Randerson J.T. (2019). Expansion of coccidioidomycosis endemic regions in the United States in response to climate change. Geohealth.

[14] Goodman R., Rauseo A.M., Windham S.L., Zuniga-Moya J.C., Powderly W.G., Spec A., Mazi P.B. (2025). Mapping the Geographic Distribution of Dimorphic Mycoses Using a US Commercial Insurance Database. Open Forum Infect. Dis..

[15] Barker B.M., Thompson G.R., Ampel N.M. (2024). Challenges to Implementing a Vaccine for Coccidioidomycosis. Open Forum Infect. Dis..

[16] Cole G.T., Xue J.M., Okeke C.N., Tarcha E.J., Basrur V., Schaller R.A., Herr R.A., Yu J.J., Hung C.Y. (2004). A vaccine against coccidioidomycosis is justified and attainable. Med. Mycol..

[17] Kirkland T.N. (2016). The Quest for a Vaccine Against Coccidioidomycosis: A Neglected Disease of the Americas. J. Fungi.

[18] Pappagianis D. (1993). Evaluation of the protective efficacy of the killed Coccidioides immitis spherule vaccine in humans. The Valley Fever Vaccine Study Group. Am. Rev. Respir. Dis..

[19] Shubitz L.F., Robb E.J., Powell D.A., Bowen R.A., Bosco-Lauth A., Hartwig A., Porter S.M., Trinh H., Moale H., Bielefeldt-Ohmann H. (2021). Δcps1 vaccine protects dogs against experimentally induced coccidioidomycosis. Vaccine.

[20] NIAID Valley Fever Research Working Group NIAID Strategic Plan for Research to Develop a Valley Fever Vaccine. National Institute of Allergy and Infectious Disease 2022. https://www.niaid.nih.gov/sites/default/files/niaid-strategic-plan-for-research-to-develop-a-valley-fever-vaccine9.9.22.pdf.

[21] Galgiani J.N., Shubitz L.F., Orbach M.J., Mandel M.A., Powell D.A., Klein B.S., Robb E.J., Ohkura M., Seka D.J., Tomasiak T.M. (2022). Vaccines to Prevent Coccidioidomycosis: A Gene-Deletion Mutant of Coccidioides Posadasii as a Viable Candidate for Human Trials. J. Fungi.

[22] Knutson K., Nguyen A., Djamba Y. Yearly Summaries of Selected Communicable Diseases in California, 2012–2020. California Department of Public Health, Infectious Diseases Branch. https://www.cdph.ca.gov/Programs/CID/DCDC/CDPH%20Document%20Library/YearlySummariesofSelectedCommDiseasesinCA2012-2020.pdf.

[23] Peterson C., Chu V., Lovelace J., Almekdash M.H., Lacy M. (2022). Coccidioidomycosis Cases at a Regional Referral Center, West Texas, USA, 2013–2019. Emerg. Infect. Dis..

[24] California Department of Public Health (2023). Coccidioidomycosis Enhanced Surveillance Questionnaire 2023.

[25] Bass J., Porter R.S. Public Health Statistics Survey Center, Opinion Research Corporation, Centers for Disease Control and Prevention. BRFSS: 2008 Health Status and Health Risk Behaviors of Arizonans. Arizona Department of Health Services. https://www.azdhs.gov/documents/preparedness/public-health-statistics/behavioral-risk-factor-surveillance/annual-reports/rpt08.pdf.

[26] Tsang C.A., Anderson S.M., Imholte S.B., Erhart L.M., Chen S., Park B.J., Christ C., Komatsu K.K., Chiller T., Sunenshine R.H. (2010). Enhanced surveillance of coccidioidomycosis, Arizona, USA, 2007–2008. Emerg. Infect. Dis..

[27] US Census Bureau PUMS Data. https://www.census.gov/programs-surveys/acs/microdata/access.html.

[28] R Core Team R: A Language and Environment for Statistical Computing. R Foundation for Statistical Computing, Vienna, Austria 2025. https://www.R-project.org/.

[29] Lumley T. (2026). Survey: Analysis of Complex Survey Samples 2026. R Package Version 4.5.

[30] Centers for Diseases Control and Prevention Influenza Vaccination Coverage for All Ages (6+ Months). Last updated 2026. https://data.cdc.gov/Flu-Vaccinations/Influenza-Vaccination-Coverage-for-All-Ages-6-Mont/vh55-3he6.

[31] Green M., Boston Children’s Hospital, Harvard Medical School, Icahn School of Medicine at Mount Sinai US Measles Vaccination Map 2025. https://abcotvdata.github.io/us-vaccination-map/.

[32] Goodwin Cartwright B.M., Masters N.B., Gilbert K.M., Rodriguez P.J., Do D., Stucky N. (2025). Early MMR Vaccine Adoption During the 2025 Texas Measles Outbreak. JAMA Netw. Open.

[33] Rencken C.A., Dunsiger S., Gjelsvik A., Amanullah S. (2020). Higher education associated with better national tetanus vaccination coverage: A population-based assessment. Prev. Med..

[34] Lu P., O’Halloran A., Ding H., Liang J.L., Williams W.W. (2016). National and State-Specific Td and Tdap Vaccination of Adult Populations. Am. J. Prev. Med..

[35] Solís Arce J.S., Warren S.S., Meriggi N.F., Scacco A., McMurry N., Voors M., Syunyaev G., Malik A.A., Aboutajdine S., Adeojo O. (2021). COVID-19 vaccine acceptance and hesitancy in low- and middle-income countries. Nat. Med..

[36] World Health Organization Behavioural considerations for acceptance and uptake of COVID-19 vaccines: WHO technical advisory group on behavioural insights and sciences for health. World Health Organization 2020. https://iris.who.int/handle/10665/337335.

[37] Wagner R., Montoya L., Head J.R., Campo S., Remais J., Taylor J.W. (2023). Coccidioides undetected in soils from agricultural land and uncorrelated with time or the greater soil fungal community on undeveloped land. PLoS Pathog..

[38] Weaver A.K., Keeney N., Head J.R., Heaney A.K., Camponuri S.K., Collender P., Bhattachan A., Okin G.S., Eisen E.A., Sondermeyer-Cooksey G. (2025). Estimating the Exposure-Response Relationship between Fine Mineral Dust Concentration and Coccidioidomycosis Incidence Using Speciated Particulate Matter Data: A Longitudinal Surveillance Study. Environ. Health Perspect..

[39] Olsen R. (2008). Self-selection bias. Encyclopedia of Survey Research Methods.

[40] Mitchell S.L., Schulkin J., Power M.L. (2023). Vaccine hesitancy in pregnant Women: A narrative review. Vaccine.

