# Supplementary material for: Intent to Accept a Valley Fever Vaccine for Humans and Dogs and Factors Influencing Intended Uptake: A Cross-Sectional Survey in Two Endemic Regions
Source: J Fungi (Basel). 2026 Jun 10;12(6):420. doi: 10.3390/jof12060420 (PMC13301879; doi:10.3390/jof12060420)
Supplement: Supplementary file 1 [file jof-12-00420-s001.zip › jof-4320829-supplementary.pdf]

Supplementary Material for

Intent to accept a Valley fever vaccine for humans and dogs and factors influencing intended uptake: a cross-sectional survey in two endemic regions

Julia N. Hermann[1,†], Sophia E. Kruger[1,†], Natalie Wodniak[1], Jammie Holland[2], Veronica Janosick[2], Asley Sanchez[2], Julio C. Zuniga-Moya[3], Dana Brucker[4], Bianca Torres[4], Keny Mendoza Melo[4], Emilse Oliveros[4], Rasha Kuran[4,5,6], Carlos D'Assumpcao[4,5,6], Royce H. Johnson[4,5,6], R. Scott Van Pelt[7], Abinash Bhattachan[8], Abram L. Wagner[1], Jennifer R. Head[1,9*]

This document includes:
Supplementary Tables S1, S2, S3
Copy of the study questionnaire
Copies of consent forms used at Kern Medical and in West Texas

Supplementary Table S1. Participant demographics in the Bakersfield (N=103) and West Texas (N=230) survey populations

	Bakersfield, CA (N=103)		West TX (N = 230)
	**n (%)**		**n (%)**
**Sex**			
Male	21 (20.4)		72 (31.3)
Female	82 (79.6)		158 (68.7)
**Age**			
18-30	26 (25.2)		43 (18.7)
31-40	29 (28.2)		49 (21.3)
41-50	24 (23.3)		45 (19.6)
51-65	19 (18.4)		49 (21.3)
66+	5 (4.9)		44 (19.1)
**Race/Ethnicity**			
Hispanic	46 (44.7)		130 (56.5)
Non-Hispanic White	38 (36.9)		82 (35.7)
Non-Hispanic Other	19		18
Non-Hispanic American Indian/Alaska Native	0 (0)		2 (0.9)
Non-Hispanic Asian/Pacific Islander	11 (10.7)		4 (1.7)
Non-Hispanic Black	3 (2.9)		8 (3.5)
Non-Hispanic Other	5 (4.9)		4 (1.7)

Supplementary Table S2. Unweighted crosstabulations of participant willingness to vaccinate self (unweighted %) by coccidioidomycosis risk factor

		Bakersfield, CA (N = 103)					West TX (N = 230)		
	n	Willing (%) (n=75)	Unsure (%) (n=21)	No (%) (n=7)		n	Willing (%) (n=99)	Unsure (%) (n=94)	No (%) (n=37)
**Overall**	103	73.8	20.4	6.8		230	43.0	40.9	16.1
**Sex**									
Male	21	85.7	9.5	4.8		72	44.3	39.9	15.8
Female	82	69.5	23.2	7.3		158	40.3	43.1	16.7
**Age**									
18-30	26	76.9	19.2	3.8		43	51.2	34.9	14.0
31-40	29	69.0	13.8	17.2		49	38.8	51.0	10.2
41-50	24	75.0	20.8	4.2		45	42.2	40.0	17.8
51-65	19	68.4	31.6	0.0		49	32.7	46.9	20.4
66+	5	80.0	20.0	0.0		44	52.3	29.5	18.2
**Race/Ethnicity**									
Hispanic	46	73.9	19.6	6.5		130	48.5	34.6	16.9
Non-Hispanic White	38	73.7	15.8	10.5		82	31.7	51.2	17.1
Non-Hispanic Other	19	68.4	31.6	0.0		18	55.6	38.9	5.6
**Immunocompromised status**									
Immunocompromised	18	72.2	22.2	5.6		48	33.3	50.0	16.7
Not immunocompromised	85	72.9	20.0	7.1		182	45.6	38.5	15.9
**Frequency of digging in soil**									
More than once a month	19	78.9	15.8	5.3		74	35.1	45.9	18.9
Less than once a month	84	71.4	21.4	7.1		154	46.8	38.3	14.9
Missing	0					2			
**Participate in leisure activities that expose to dust**									
More than once a month	76	71.1	21.1	7.9		159	40.9	44.0	15.1
Less than once a month	27	77.8	18.5	3.7		67	46.3	35.8	17.9
Missing	0					4			

**Caught in a dust storm**									
Yes	63	68.3	25.4	6.3		171	41.5	44.4	14.0
No	32	87.5	6.3	6.3		46	47.8	28.3	23.9
Unsure	8	50.0	37.5	12.5		9	44.4	44.4	11.1
Missing	0					4			
**Ever had an outdoor occupation**									
Yes	17	88.2	11.8	0.0		56	35.7	50.0	14.3
No	86	69.8	22.1	8.1		172	44.8	38.4	16.9
Missing	0					2			
**Awareness of Valley fever**									
Previously aware						40	62.5	27.5	10.0
Previously unaware						188	39.4	43.1	17.6
Unsure						2	0	0	100

Supplementary Table S3. Unweighted crosstabulations of participant willingness to vaccinate their dog (unweighted %) by coccidioidomycosis risk factor

		**Bakersfield, CA (N=78)**					**West TX (N = 178)**		
	**n**	**Willing (%) (n=56)**	**Unsure (%) (n=21)**	**No (%) (n=1)**		**n**	**Willing (%) (n=91)**	**Unsure (%) (n=59)**	**No (%) (n=28)**
**Overall**	78	71.8	26.9	1.3		178	51.1	33.1	15.7
**Sex**									
Male	15	73.3	26.7	0.0		55	49.1	36.4	14.5
Female	63	71.4	27.0	1.6		123	52.0	31.7	16.3
**Age**									
18-30	18	66.7	33.3	0.0		32	56.3	28.1	15.6
31-40	24	70.8	25.0	4.2		42	52.4	35.7	11.9
41-50	22	77.3	22.7	0.0		34	55.9	26.5	17.6
51-65	10	60.0	40.0	0.0		42	45.2	35.7	19.0
66+	4	100.0	0.0	0.0		28	46.4	39.3	14.3
**Race/Ethnicity**									
Hispanic	37	73.0	24.3	2.7		90	53.3	25.6	21.1
Non-Hispanic White	31	74.2	25.8	0.0		77	46.8	44.2	9.1
Non-Hispanic Other	10	60.0	40.0	0.0		11	63.6	18.2	18.2
